# Microfacies Characteristics of Late Pennsylvanian Cyclothems on the Carbonate Platform Margin in Guizhou, South China

**DOI:** 10.3390/life14111495

**Published:** 2024-11-16

**Authors:** Junjie Wang, Enpu Gong, Yongli Zhang, Xiao Li, Lifu Wang, Guanming Lai, Depeng Li

**Affiliations:** 1 College of Resources and Civil Engineering, Northeastern University, No. 3-11, Wenhua Road, Shenyang 110819, China; 1910350@stu.neu.edu.cn (J.W.); 1710351@stu.neu.edu.cn (X.L.); 2110395@stu.neu.edu.cn (G.L.); 2010380@stu.neu.edu.cn (D.L.); 2 School of Civil Engineering, Liaoning Petrochemical University, Fushun 113001, China; wanglifu@lnpu.edu.cn

**Keywords:** cyclothems, microfacies, reefs, late Paleozoic ice age, South China

## Abstract

Late Pennsylvanian cyclothems are documented from the carbonate platform margin in Guizhou, South China, providing a unique opportunity to study glacio-eustatic fluctuations and their impact on reef development. This paper focuses on a shallow-water, reef-bearing succession and a deep-water succession in the Houchang area of Guizhou. Fourteen microfacies, grouped into seven associations, represent distinct depositional environments. These microfacies associations exhibit vertical cyclicity, interpreted as cyclothems, similar to those observed globally, which are attributed to the waxing and waning of the Gondwana ice sheet. The cyclothems are primarily composed of sediments below the wave base within a shallow-water platform margin and deep-water settings. Those cyclothems show strong correlations with those observed in South China, Ukraine, and the North American Midcontinent, suggesting a potential connection to global glacio-eustatic processes. A brief and rapid sea-level rise during the late Kasimovian may correspond to a recently recognized global warming event. A microfacies analysis indicates that these cyclothems reflect glacial-type sea-level fluctuations ranging from 15 to 35 m. Notably, the reef-bearing cyclothems correspond to intermediate, major cyclothems identified in South China and the Midcontinent from the late Moscovian to early Kasimovian stages. The global cyclothem correlations and reef development patterns in South China suggest that intermediate, major cycles were the primary controls on reef growth and demise, while minor cycles influenced biostromes and community succession within the reefs. These findings underscore the pivotal role of the Late Paleozoic Ice Age (LPIA) in shaping reef development in far-field regions during the Late Pennsylvanian.

## 1. Introduction

The Late Paleozoic Ice Age (LPIA), spanning from the end-Devonian to the early Permian (~100 Ma), represents a significant icehouse period [1,2,3,4,5,6,7,8,9,10,11,12,13,14,15]. Substantial evidence indicates that the LPIA was composed of multiple deglaciation and glaciation events [1,5,8,16]. The Late Pennsylvanian marks the peak of the LPIA [3,17,18], distinguished by a cold climate and widespread ice sheets in high paleolatitude regions [1,3,5,19,20,21,22,23,24,25]. These conditions induced significant high-frequency sea-level fluctuations on a global scale [12,26,27,28,29,30,31,32], resulting in the development of cyclothems in far-field regions.

The study and correlation of cyclothems have largely focused on platform and slop settings, such as those found in the North American Midcontinent, Ukraine, and South China [27,29,30]. In contrast, platform margin settings have received relatively less attention. These carbonate platform margins are crucial sites for bioconstruction and carbonate production, acting as sensitive indicators of climatic and sea-level changes [33,34,35,36,37]. Thus, further research into the sedimentary characteristics of platform margins is warranted.

During the Pennsylvanian, the Guizhou region remained tectonically stable [38]. Consequently, the high-frequency depositional sequences observed in this interval are primarily attributed to glacio-eustatic sea-level fluctuations [30,31]. The Houchang area in Guizhou exemplifies platform margin deposits characterized by well-documented bioconstructions [33,37,39,40,41,42,43,44], while the Zhongdi section in Guizhou offers a comparative reference for cyclothem analysis.

This study investigates both shallow- and deep-water successions along the platform margin in the Houchang area, Guizhou, South China. By conducting detailed lithological analyses, reconstructing depositional environments, and comparing cyclothems with those in the Zhongdi area and the Midcontinent, this research aims to clarify the far-field response of platform margin settings to the Late Paleozoic Ice Age.

## 2. Geological Setting

### 2.1. Palaeogeography

**Figure 1:**
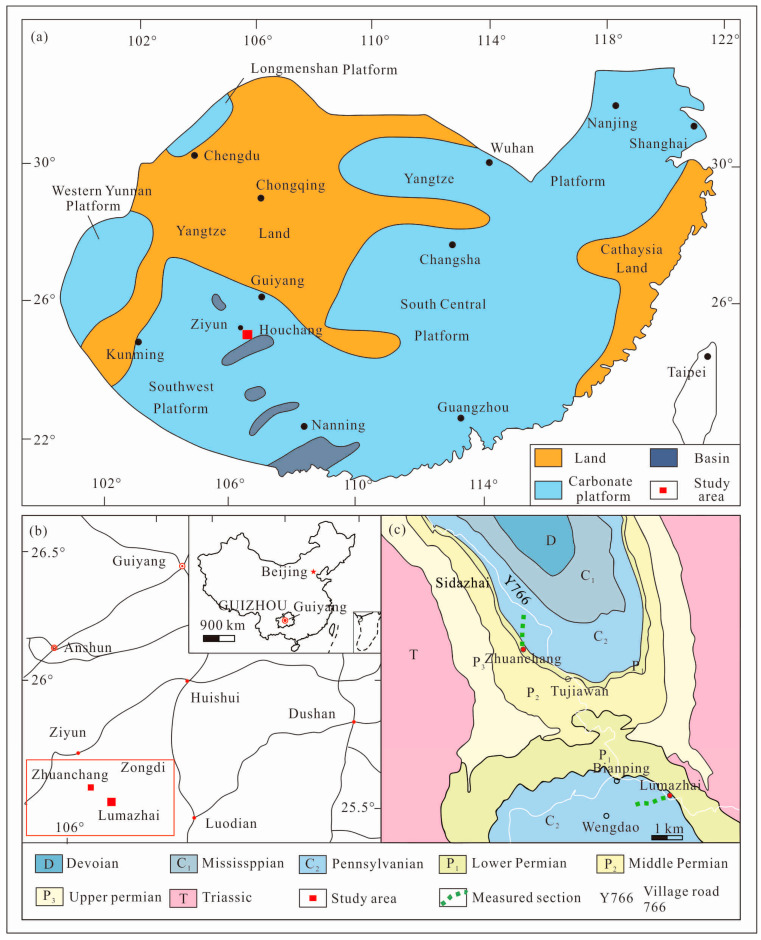
General geological information of this study: (**a**) The Late Pennsylvanian paleogeography of South China (after [38]). (**b**) Location of the Houchang study area in Guizhou Province (map modified after the Standard Map Service of the National Administration of Surveying). (**c**) Geological map of the Houchang study area.

During the Late Pennsylvanian, the South China Block was situated to the east of the Palaeo-Tethys Ocean in the low-latitude regions. This block consisted of lands, shallow carbonate platforms, and basins [38]. Guizhou is situated on the southern margin of the Yangtze Plate and belongs to the stable tectonic region of the Dian-Qian-Gui platform [31,39]. The area is influenced by two prominent sets of fractures oriented northwest and northeast, which have facilitated the development of isolated carbonate platforms of varying sizes within the deep-water basin, spanning from the late Early Devonian to the Permian [39]. The study area is located in Houchang town, Ziyun County, Guizhou, South China (Figure 1), on a southwest carbonate platform at the northern margin of the Luodian intraplatform basin. The sequence stratigraphy is continuous and consists of Late Devonian to Early Permian strata [38] (Figure 1). The area is influenced by two prominent sets of fractures oriented northwest and northeast, which have facilitated the development of isolated carbonate platforms of varying sizes within the deep-water basin, spanning from the late Early Devonian to the Permian. Between these isolated platforms, deep-water shelf and platform facies sediments are commonly found [33,40,41,42,43].

### 2.2. Biostratigraphy

#### 2.2.1. Lumazhai Section

**Figure 2:**
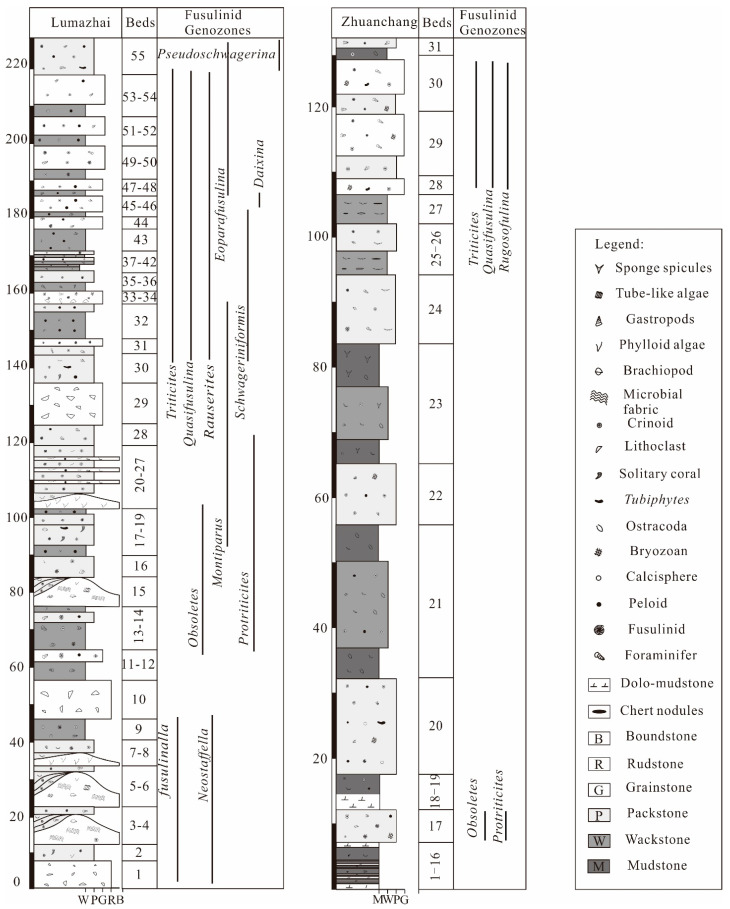
Facies and fusulinid zone in topmost Moscovian–Gzhelian stratigraphic column of Zhuanchang and Lumazhai sections.

The Lumazhai section (25°30′27.0″ N, 106°14′03.0″ E) is situated northwest of Lumazhai village and is 226 m in thickness (Figure 2). Common coral fossils, primarily comprising *Fomichevella* and *Ivanovia*, are observed in the section. In some beds (2, 18, and 30), *Fomichevella* shows a scattered distribution, occasionally accompanied by *Ivanovia*. Beds 35, 37, 39 and 41 display a prolific abundance of *Fomichevella*, forming expansive coral biostromes [44]. *Fomichevella*-dominated coral fossil assemblages exhibit distinct characteristics indicative of the Pennsylvanian [45].

**Figure 3:**
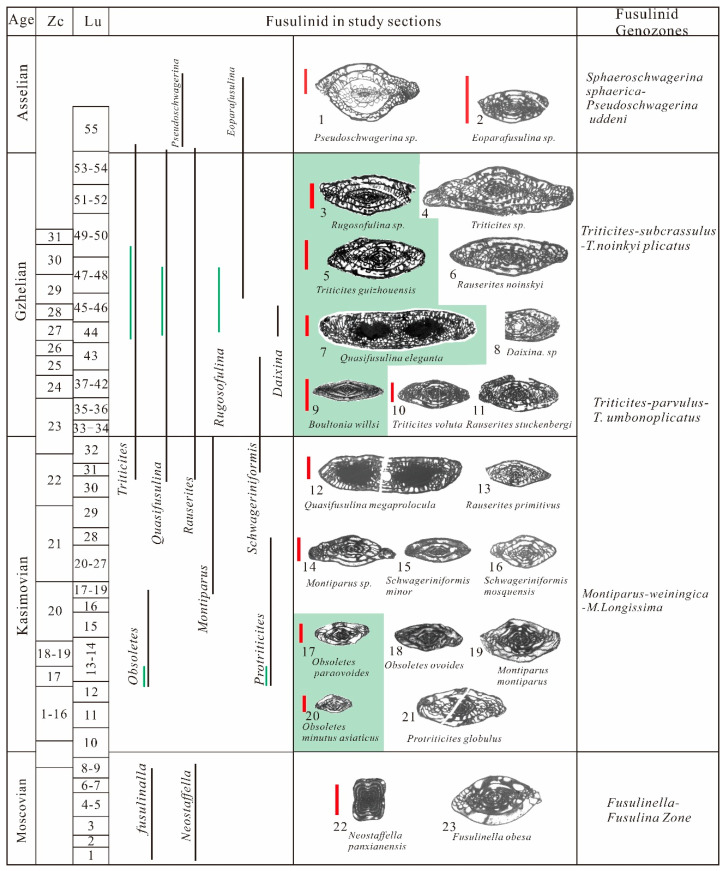
Stratigraphic framework for the study sections with a focus on the revised fusulinid biostratigraphy. Zc = Zhuanchang section, Lu = Lumazhai section, Vertical scale red bars for fusulinids are all 1 mm, Fusulinas of Zhuanchang in light green area. No fusulinid in beds 18–27 in Zhuanchang section. 1—*Pseudoschwagerina* sp.; 2—*Eoparafusulina* sp.; 3—*Rugosofulina* sp.; 4—*Triticites* sp. 5—*Triticites* sp.; 6—*Rauserites noinskyi* [52]; 7—*Quasifusulina eleganta* [53]; 8—*Daixina*. sp.; 9—*Boultonia willsi* [54]; 10—*Triticites volute* [55]; 11—*Rauserites stuckenbergi* [52]; 12—*Quasifusulina megaprolocula* [56]; 13—*Rauserites primitivus* [57]; 14—*Montiparus* sp.; 15—*Schwageriniformis minor* [57]; 16—*Schwageriniformis mosquensis* [57]; 17—*Obsoletes Paraovoides* [58]; 18—*Obsoletes ovoides* [59]; 19—*Montiparus montiparus* [60]; 20—*Obsoletes minutus* var. *asiatica* [61]; 21—*Protriticites globulus* [62]; 22—*Neostaffella Panxianensis* [47]; 23—*Fusulinella obesa* [46].

Fusulinids are abundant and widely distributed throughout the section, providing valuable tools for establishing biostratigraphic frameworks (Figure 3). The prevalence of *Fusulinella* and *Neostafflla* in beds (1–9), characterized by the abundance of *Neostaffella Panxianensis* and *Fusulinella obesa*, indicates the late Moscovian Stage [46,47,48]. Beds 10 and 11 exhibit a relatively sparse occurrence of fusulinids, and its stage remains uncertain. Beds 12–20 are dominated by the coexistence of *Obsoletes* and *Protriticites*. *Montiparus* first occur in bed 17, albeit in low abundance. The fusulinid assemblage can be effectively correlated with the common *Protriticites*-*Obsoletes* zone of the early Kasimovian Stage [49,50]. Beds 20–27 are dominated by the coexistence of *Montiparus* and *Protriticites* and *Montiparus*.

Fusulinids are notably scarce in bed 29, characterized by only a few occurrences of *Montiparus*. This scarcity suggests a plausible correlation with the middle *Montiparus* acme zone of the Kasimovian Stage [49]. Beds 30–31 record the first occurrence of *Rauserites*, *Triticites*, and *Schwageriniformis*, with *Schwageriniformis* proliferating significantly. Additionally, this interval contains a considerable number of *Montiparus*, corresponding to the late Kasimovian Stage [49].

Beds 32–43 are characterized by a predominant assemblage of abundant *Triticites*, *Quasifusulina*, and *Rauserites*. Additionally, the first occurrences of *Triticites rarvulus* and *Rauserites stuckenbergi* indicate the early Gzhelian Stage according to fusulinid assemblage in western Guizhou [48]. Beds 44–54 are dominated by *Rauserites*, *Triticites*, *Rugosofulina*, and *Quasifusulina*, with the first occurrence of *Daixina* in beds 45 and 46. This assemblage can be correlated with the *Triticites subcrassulus*-*T*. *noinkyi plicatu* zone, indicating the late Gzhelian Stage [48]. *Eoparafusulina* and *Pseudoschwagerina* are found in bed 55, representing the fusulinid assemblage of the *Sphaeroschwagerina sphaerica*-*Pseudoschwagerina uddeni* zone and indicating the Asselian Stage [48].

#### 2.2.2. Zhuanchang Section

The Zhuanchang section (25°33′15.2″ N, 106°11′50.1″ E) is situated near the Zhuanchang villages and is 126 m in thickness (Figure 2). The distribution of fusulinids is primarily concentrated at the bottom (bed 17) and the top (beds 28 and 30). The genera *Obsoletes* and *Protriticites* commonly occur in bed 17, which is similar to characteristics of the *Protriticites*-*Obsoletes* zone of the early Kasimovian Stage [49,50]. Additionally, the common occurrences of *Obsoletes paraovoides* further supports this viewpoint [51]. Beds 28 and 30 contain *Quasifusulina*, *Rugosofusulina*, *Triticites, Psedofusulina,* and *Boultonia*. The appearance of *Rugosofusulina*, *Psedofusulina,* and *Boultonia* is indicative of a late Gzhelian Stage, which likely corresponds to the *Triticites subcrassulus*-*T. noinkyi plicatu* zone [48].

## 3. Materials and Methods

The two carbonate sections were measured and described in terms of lithology, color, bed thickness, sedimentary structures, and fossil content. These characteristics were utilized to identify sedimentary cycles within the successions. A total of 200 thin sections, 200 biological thin sections, and 16 polished slabs were prepared for sedimentary environment analysis. Microfacies analysis followed Flügel’s (2010) methodology [63], utilizing polarized light microscopy to assess lithology, texture, grain types, and biotic assemblages. Carbonate classification followed the structural frameworks of Dunham (1962) and Embry and Klovan (1971) [64,65].

## 4. Results

Fourteen microfacies (Table 1) have been identified and grouped into seven microfacies associations across the Lumazhai and Zhuanchang sections: (1) platform margin slope (A), (2) reef environment within the platform margin (B), (3) back-reef environment within the platform margin (C), (4) margin shoal environment (D), (5) deep-water environment (E), (6) low-energy shallow-water environment, and (7) moderate to high-energy shallow-water environment.

### 4.1. Microfacies Analysis in Lumazhai Section

#### 4.1.1. Microfacies Association A

**Figure 4:**
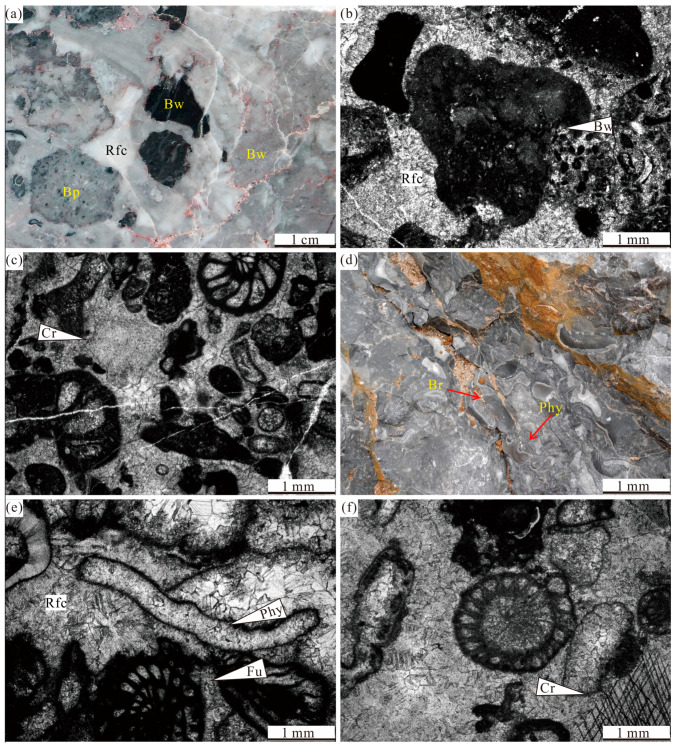
Representative facies of Lumazhai section: (**a**) Polished slab photograph of intraclastic rudstone at ~7 m. (**b**,**c**) Photomicrograph of intraclastic rudstone, containing foraminifers, crinoids, algae. (**d**) Field photograph of bioclastic floatstone–rudstone at~128 m, containing phylloid algae, brachiopods. (**e**,**f**) Photomicrograph of bioclastic floatstone–rudstone, containing phylloid algae, fusulinid, crinoids. Bp—bioclastic packstone; Rfc—radial fibrous cement; Br—brachiopod; Bw—bioclastic wackestone; Fu—fusulinid; phy—phylloid algae and Cr—crinoid.

Microfacies association A is primarily distributed in bed 1 and consists mainly of intraclastic rudstone (F1). Intraclasts mainly include bioclastic wackestone–packstone–grainstone, phylloid algal boundstone, and microbial boundstone. These intraclasts are angular and vary in size, ranging from 0.5 to 7 cm. The spaces between the intraclasts are predominantly filled with blocky and radial-fibrous cements (Figure 4a–c).

Interpretation: The poorly sorted and subrounded intraclasts suggest that they were transported over a short distance before deposition. Various intraclast components are interpreted to platform margin sediments. Intraclast-supported and mud-free texture suggests relatively good water circulation. Therefore, microfacies association A likely represents a platform margin slope setting [43,66].

#### 4.1.2. Microfacies Association B

Microfacies association B consists of bioclastic floastone–rudstone (F2), bioclastic packstone–grainstone (F3), phylloid algal boundstone (F4), and microbial boundstone (F5).

**Figure 5:**
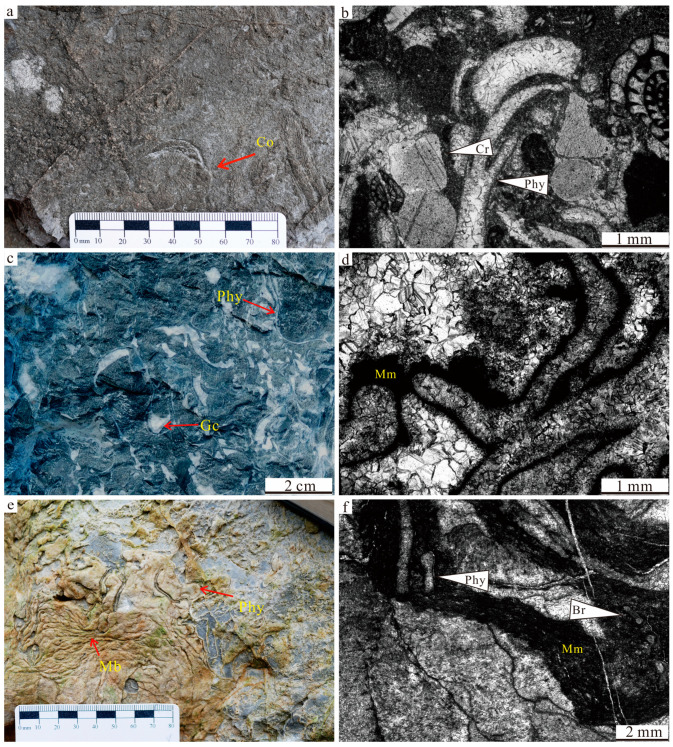
Representative facies of Lumazhai section: (**a**) Field photograph of coral in bioclastic packstone. (**b**) Photomicrograph of bioclastic packstone, containing foraminifers, algal fragments, and crinoids. (**c**) Field photograph of phylloid algal boundstone at ~103 m. (**d**) Photomicrograph of phylloid algal boundstone, containing phylloid algae. (**e**) Field photograph of microbial carbonates at ~25 m. (**f**) Photomicrograph of microbial boundstone, containing phylloid algae, bryzoans. Co—Coral; Cr—Crinoid; Phy—Phylloid algae; Mb—Microbial boundstone; Gc—growth cavity; Co—Coral; and Mm—microbial micrite.

The debris in F2 primarily consists of phylloid algal fragments (thalli ranging from 3 to 8 cm in size), corals and brachiopod shells. Intergranular porosity is filled with blocky and radial-fibrous cements (Figure 4d–f). F2 and F3 are characterized by diverse skeletal and poorly sorted biogenic components. These components include foraminifers, algal fragments, bryozoan fragments, brachiopods, and corals (Figure 5a,b).

F4 are mainly distributed in beds 7 and 20. The phylloid algal reefs are about 2.5 and 4 m in thickness, respectively, with an indeterminate width (>4 m). Thalli are curved and elongated (8–15 cm), forming a robust framework. The internal structure of the thalli is indistinguishable due to overprinting by later diagenetic processes. The spaces between the thalli are filled with a micritic matrix and blocky and radial-fibrous cements. The biogenic components mainly consist of a few foraminifers and brachiopods (Figure 5c,d).

F4 are mainly distributed in beds 7 and 20. The phylloid algal reefs are about 2.5 and 4 m in thickness, respectively, with an indeterminate width (>4 m). Thalli are curved and elongated (8–15 cm), forming a robust framework. The internal structure of the thalli is indistinguishable due to overprinting by later diagenetic processes. The spaces between the thalli are filled with micritic matrix, blocky and radial-fibrous cements. The biogenic components mainly consist of a few foraminifers and brachiopods (Figure 5c,d).

F5 is distributed in beds 3, 5 and 15. The bed 3 microbial reef is about 9 m in thickness and extends more than 30 m in width [42]. The bed 5 microbial reef is about 10 m high with an indeterminate width. The bed 15 microbial reef is about 9.5 m in thickness and with an indeterminate width. Microbial boundstone is formed by microbial binding organisms and self-calcification (Figure 5e,f). The main associational organisms include algae, corals, and bryozoans [42,43].

Interpretation: poorly sorted and angular debris in F2 indicate in situ component. The assemblage of corals, foraminifers, green algae, and brachiopods, represents a photozoan association in a shallow open marine environment [63]. Abundant marine cement and grain-supported fabric suggests good water circulation [43,67]. The well-preserved algal thalli and cavities filled with radial-fibrous cements suggest that reef formed in situ. According to Gong et al. (2007a) [33], the paleoecology of phylloid algal reef indicates that they typically inhabit areas below the fair-weather wave base, experiencing moderate hydrodynamic conditions within the euphotic zone. Microbial reefs are formed in a platform margin to an upper slope environment with active water circulation and within the euphotic zone [43]. Collectively, microfacies association B represents a reef environment in a platform margin.

#### 4.1.3. Microfacies Association C

Microfacies association C consists of bioclastic wackestone–packstone (F6) and intraclastic breccia (F7).

**Figure 6:**
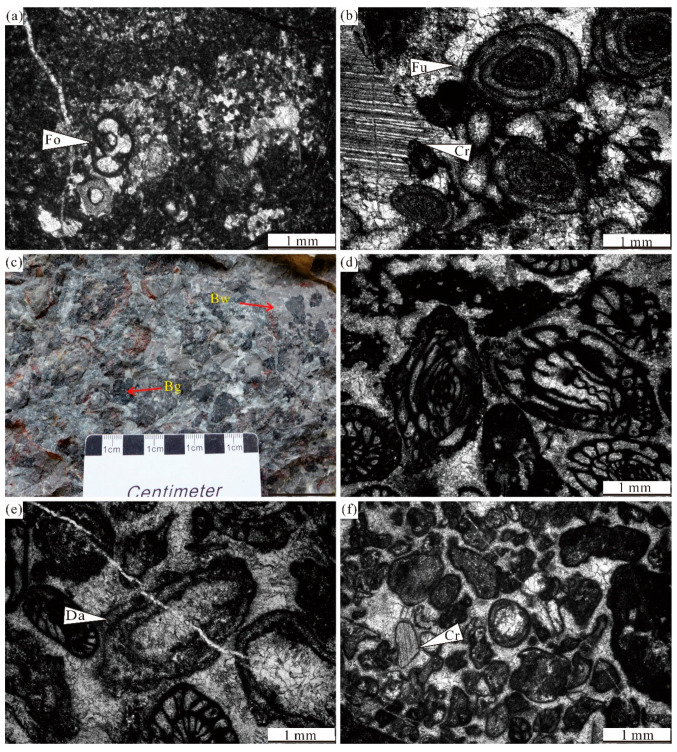
Representative facies of Lumazhai section: (**a**) Photomicrograph of bioclastic wackestone, containing foraminifers, and crinoids. (**b**) Photomicrograph of bioclastic packstone, containing foraminifers, crinoids, and algal fragments. (**c**) Field photograph of intraclastic breccia at ~156 m. (**d**) Photomicrograph of grainstone in intraclastic breccia, containing fusulinids. (**e**) Photomicrograph of bioclastic grainstone, containing fusulinids, foraminifers, and dasyclad green algae. (**f**) Photomicrograph of coated bioclastic grainstone, containing foraminifers, crinoids, and algal fragments. Fo—foraminifer; Bw—bioclastic wackestone; Bg—bioclastic grainstone; Cr—crinoid; Fu—fusulinid; and Da—Dasyclad green algae.

F6 are common in the Lumazhai area. The biogenic components have low biodiversity and are characterized by foraminifers, fusulinids, bivalves, crinoids, and algae. *Staffella* is common in this microfacies. Bioturbation is common with internal filling by foraminifers and crinoids (Figure 6a,b). Additionally, peloids are common, exhibiting fuzzy margins and subrounded shapes, ranging in size from 40 to 80 μm.

F7 are mainly distributed in bed 34, with 0.4 m in thickness. The intraclasts are primarily composed of dark-gray bioclastic grainstone (ranging from 0.5 to 2 cm in size), which make up approximately 60–70% of the composition in volume (Figure 6c,d). The spaces between the angular fragments are filled with bioclastic wackestone (F6).

Interpretation: The fuzzy and variable sizes of peloids suggest that these peloids formed in situ due to microbial activity in a low-moderate water condition [63,68]. The association of algae and foraminifers indicates a shallow-water environment within the photic zone. The common presence of *Staffella* is likely associated with restricted environments [69]. The angular and poorly sorted intraclastics in F7 suggest in situ deposition or a proximal setting. The abrasive skeletal grains and mud-free texture in the grainstone indicate they formed in a water wash, high-energy shoal environment [63]. Combining with bioclastic wackestone (F6), F7 is interpreted as a low-energy environment influenced by storms. Collectively, Microfacies association C is interpreted as protected back-reef environment in the platform margin.

#### 4.1.4. Microfacies Association D

The microfacies association D is common in Lumazhai area. It consists of bioclastic grainstone (F8) coupled with coated bioclastic grainstone (F9). The grains in F8 are mainly composed of fusulinids, foraminifers, crinoids, *Tubiphytes*, bryozoans, brachiopods, and algal fragments. The grains in F9 are mainly composed of subrounded to ellipsoid crinoids, ranging from 0.3 to 2.5 mm in size. The coated grains include fusulinids, algae, foraminifers, and crinoids. Intergranular porosity in those microfacies is filled with blocky calcite and radial fibrous cements (Figure 6e,f).

Interpretation: the grainstone texture and abundant marine cement suggest high hydrodynamic conditions [63]. The skeletal association of foraminifers, fusulinids, and green algae represents a photozoan assemblage in a shallow marine setting. Dasycladaceans are common in very shallow environments. Additionally, coated grains with a micrite envelope are interpreted as forming underwater flush conditions with microbial activity, common in shoal environments [63,70]. Therefore, microfacies association D represents a shoal environment with turbulent waters above the fair-weather wave base.

### 4.2. Microfacies Analysis in Zhuanchang Section

#### 4.2.1. Microfacies Association E

This microfacies association E consists of black, 15–45 cm thick fine-grain burrowed wackestone (Z1) and spiculitic wackestone (Z2).

**Figure 7:**
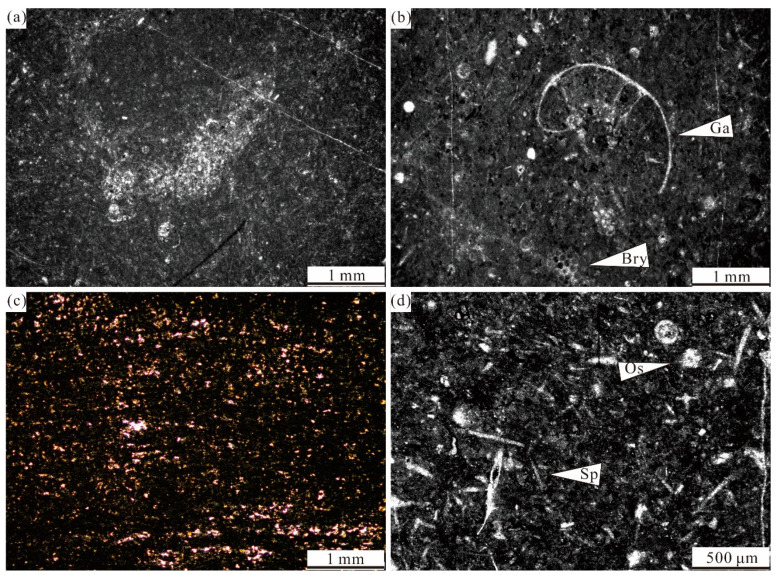
Representative facies of Zhuanchang section: (**a**,**b**) Photomicrograph of fine-grain burrowed wackestone with burrow, containing peloids, bryzoans, ostracods, and gastropod. (**c**) Photomicrograph of dolomite limestone with 50–100 μm of dolomite. (**d**) Photomicrograph of spiculitic wackestone, containing sponge spicules, ostracods, and peloids. Ga—gastropod; Bry—bryozoan; Os—ostracod; and Sp—sponge spicule.

The grains in Z1 are mainly composed of peloids and a few sponge spicules, small bivalve shells, bryozoans, and thin-shelled gastropods. The burrows are common and filled by echinoderm fragments (Figure 7a,b). Locally, Z1 is locally dolomized (Figure 7c). The grains in Z2 primarily consist of angular sponge spicules in size ranging from 0.3 to 0.8 mm, peloids, bivalves, and ostracods (Figure 7d).

Interpretation: the poorly sorted, angular sponge spicule fossils in situ or near in situ accumulation. The assemblage of spicule sponges, ostracods, and fine-peloids is common in deep environments in basin or slope settings [63,71,72]. Combined common burrows in rock, microfacies association E is thought to represent deep-water environment (70).

#### 4.2.2. Microfacies Association F

This microfacies association consists of dark-gray, 20–50 cm thick mm-scale bedded bioclastic wackestone (Z3), and encrusting foraminifer wackestone (Z4).

**Figure 8:**
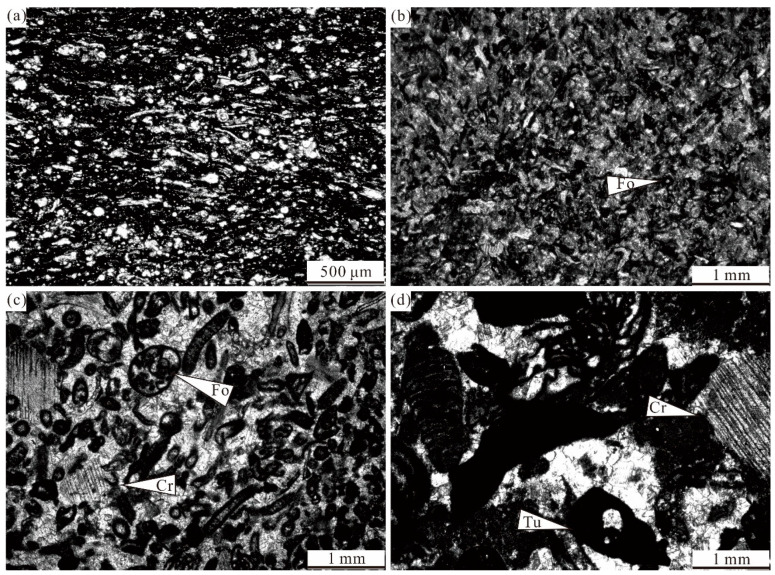
Representative facies of Zhuanchang section: (**a**) Photomicrograph of mm-scale bedded bioclastic wackestone, containing small foraminifers, ostracods, and bivalves. (**b**) Photomicrograph of encrusting foraminifer wackestone, containing small foraminifers, algal fragments, crinoids, bivalves. (**c**,**d**) Photomicrograph of bioclastic packstone–grainstone, containing foraminifers, crinoids, bryzoans, fusulinids, algal fragments, and *Tubiphytes*. Fo—foraminifer—Cr—crinoid; and Tu—*Tubiphytes*.

The skeletal grains in Z3 are primarily composed of bivalves, ostracods, crinoids, small foraminifers, and bryozoan fragments. Z3 exhibits a horizontal orientation and forms millimeter-scale bedding (Figure 8a). The skeletal grains in Z4 are primarily composed of small foraminifers and algal fragments. Bivalves, crinoids, and peloids are common (Figure 8b).

Interpretation: Z3 with horizontally oriented grains suggests a deposition controlled by weak bottom currents [56,73]. The skeletal grain assemblage, similar to Z4, is characterized by small foraminifers, ostracods, and crinoids, which are common in shallow-water environments within the photic zone [63]. The wackestone texture and poorly sorted grains indicate that Z4 represents an in-place deposition in a low-energy environment. Collectively, microfacies association F represents a shallow environment with weak water conditions within the photic zone.

#### 4.2.3. Microfacies Association G

Microfacies association G consists of dark-gray, 40–80 cm thick bioclastic packstone–grainstone facies (Z5). The biogenic components are composed of forminifers, fusulinid, bryzoans, algal fragments, bivalves, echinoderms, *Tubiphytes,* and gastropods. Peloids and mudclasts are scarce. Locally, there is green algae concentrate. Intergranular porosity is filled with a micritic matrix and blocky cement (Figure 8c,d).

Interpretation: the grain-supported texture and common blocky cement suggest moderate to high hydrodynamic conditions. High biodiversity is typically indicative of a shallow, open marine environment within the photic zone [63]. Therefore, the microfacies association G represents a shallow-water environment with moderate to high energy conditions [63].

### 4.3. Recognition and Overview of Cyclothems

Sedimentary cycles in the study area were identified through variations in microfacies associations (i.e., sedimentary environment changes). Two subtidal cycle types were recognized, including Type A (shallow-subtidal cycle) and Type B (deep-subtidal cycle). All cyclothems mainly exhibit shallowing-upward microfacies arrangements.

**Figure 9:**
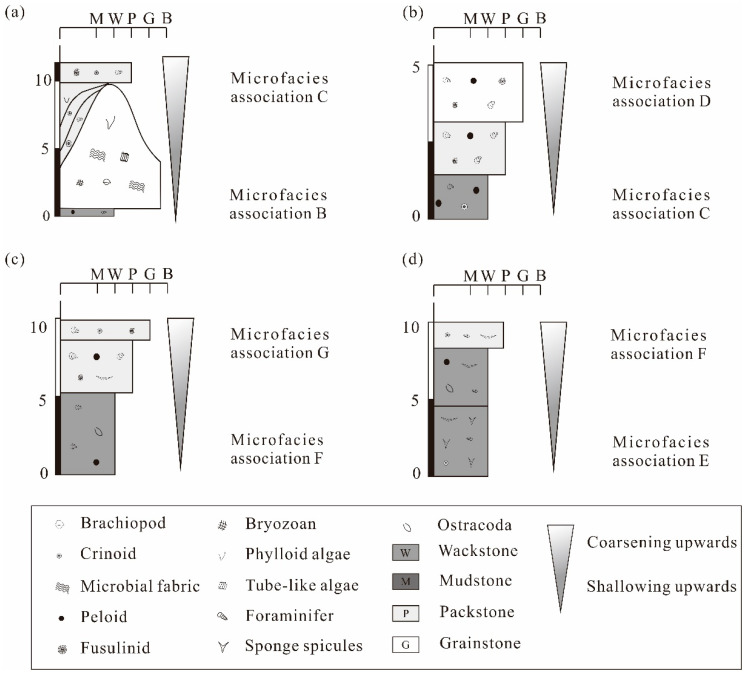
Typical sedimentary cycles showing shallowing-upward facies arrangements in Lumazahi and Zhuanchang sections: (**a**) Cyclical deposition of Type A1 in Lumazahi section. (**b**) Cyclical deposition of Type A2 in Lumazahi section. (**c**) Cyclical deposition of Type A3 in Zhuanchang section. (**d**) Cyclical deposition of Type B in Zhuangchang section.

**Figure 10:**
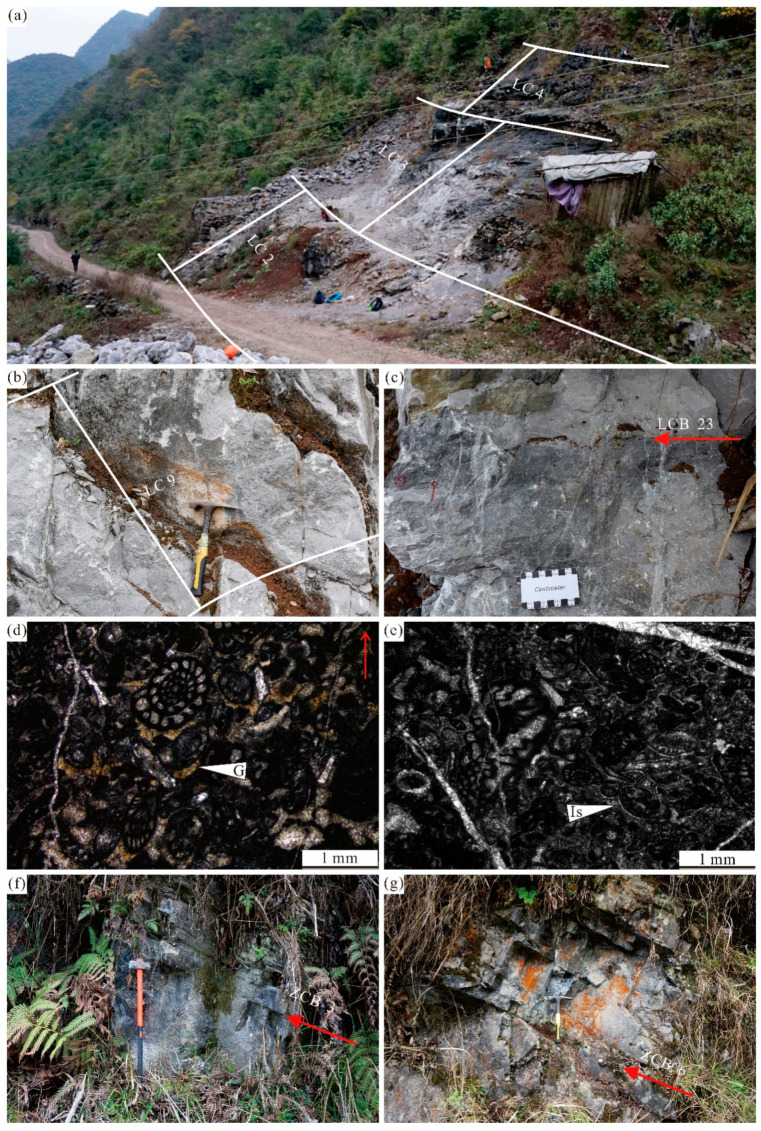
Photographs showing typical sedimentary cycles observed in Lumazhai and Zhuanchang sections: (**a**) Field photograph of Type A1 (reef-bearing cycle), showing LC (1–3) in the Lumazhai section. (**b**) Field photograph of Type A1, showing phylloid algal floatstone–bioclastic (lower part)–grainstone (upper part) arrangement. (**c**) Field photograph of cycle boundary of Type A2, showing dark bioclastic grainstone (lower part) with pendant cement and gray bioclastic wackstone (upper part). (**d**) Photomicrograph of bioclasic grainstone with pendant cement, suggesting direction of gravity at time of cement formation in a vadose environment (opposite to upwards direction of strata; red arrow). (**e**) Grains are surrounded by thin isopachous cement (Formed in a meteoric phreatic environment) in cycle 22. (**f**) Field photograph of cycle boundary of Type A3, showing bioclastic packstone–grainstone (Z5) (lower part) and mm-scale bedded bioclastic wackestone (Z3) (upper part). (**g**) Field photograph of cycle boundary of Type B, showing spiculitic wackestone (Z2) and encrusting foraminifer wackestone (Z4) (upper part).

Type A1: These cycles are characterized by the stacking of microfacies associations B and C (Figure 9a and Figure 10a). The arrangement of these microfacies indicates a transition from a reef environment to a back-reef shallow-water environment. These cycles typically range from 5 to 8 m in thickness and are predominantly observed during the late Moscovian to early Kasimovian stages.

Type A2: These cycles are formed by the stacking of microfacies associations C and D. Shallow-water intervals are marked by packstone–grainstone with pendant cement (Figure 10). Observations, combined with the absence of calcareous weathering crusts, suggest a deposition in a very shallow-water environment influenced by meteoric precipitation. The microfacies arrangement reflects a transition from a back-reef shallow-water environment to a shallow-water margin shoal environment. These cycles generally range from 1.5 to 20 m in thickness and are predominantly found in the Gzhelian Stage.

Type A3: These cycles result from the stacking of microfacies associations F and G (Z5) (Figure 9c and Figure 10f). This type is interpreted as a transition from a low-energy shallow environment to a mid- to high-energy shallow environment. The cycles typically range from 10 to 15 m in thickness and are predominantly present during the early Kasimovian and late Gzhelian stages.

Type B: These cycles are characterized by the stacking of microfacies associations E and F (Figure 9d and Figure 10g). The biotic assemblage transitions upward from a heterotrophic deep-water community (including sponge spicules and bivalves) to a phototrophic community (comprising small foraminifers, bryozoans, and algae). This type indicates a transition from a deep-water environment to a deeper, low-energy environment. These cycles generally range from 15 to 20 m in thickness and are present from the Kasimovian to early Gzhelian stages.

## 5. Discussion

### 5.1. Depositional Environment

The Houchang area is characterized by shallow, blocky bioclastic limestone, abundant in reefs, mounds, and shoals. These characteristics indicate a shallow-water platform margin distanced from the continent [33,34,42,43,44,70,74,75,76]. The microfacies association A consists of angular to subangular intraclasts originating from the platform margin, indicative of a slope environment [43,77,78]. Microfacies association B includes boundstone, bioclastic floatstone, rudstone, packstone, and grainstone. Biogetic components exhibit a high diversity. Abundant marine cements indicate good water circulation. Collectively, it represents a reef environment in the platform margin [43,70]. The microfacies association C is characterized by low diversity. The biogetic component contains foraminifers, algal fragments, peloids, and ostracods, along with common *Staffella*. Combined with poorly sorted grains, it is interpreted as a protected back-reef environment in platform margin. Microfacies association D is characterized by predominantly broken skeletal grains and coated grains. Intergranular porosity is filled by radial fibrous and blocky cements. It represents a margin shoal environment [63].

**Figure 11:**
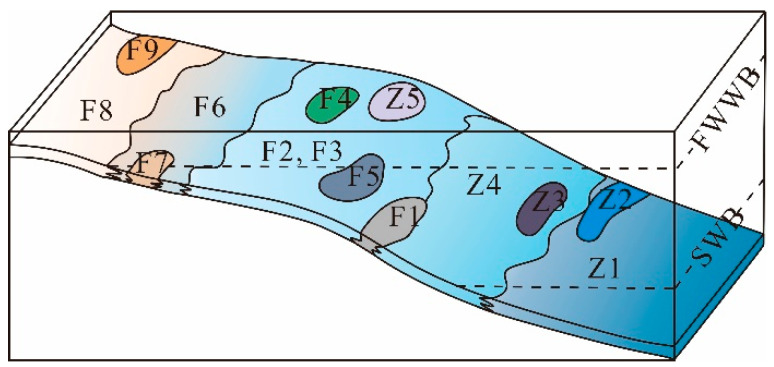
The depositional model for the Lumazhai and Zhuangchang sections, showing the distribution of components in different facies types and depositional environments: FWWB and SWWB stand for the fair-weather wave base and storm-weather wave base, respectively, after [70]. F1—Intraclastic rudstone facies; F2—Bioclastic floatstone–rudstone facies; F3—Bioclastic packstone–grainstone facies; F4—Phylloid algal boundstone facies; F5—Microbial boundstone facies; F6—Bioclastic wackestone–packstone facies; F7—Intraclastic breccia facies; F8—Bioclastic grainstone facies; F9—Coated bioclastic grainstone facies; Z1—Fine-grain burrowed wackestone facies; Z2—Spiculitic wackestone facies; Z3—Mm-scale bedded bioclastic wackestone facies; Z4—Encrusting foraminifer wackestone facies; and Z5 Bioclastic packstone–grainstone facies.

The microfacies association E is mainly composed of abundant lime with common burrows and a heterotrophic assemblage, indicating a deep-water environment. Microfacies association F is characterized by phototrophic assemblage, which is composed of small foraminifers, ostracods, algae, bivalves, and crinoids. Combined with wackestone texture and poorly sorted grains, this association represents a shallow environment with weak water conditions within the photic zone. The microfacies association G is characterized by a high-diversity phototrophic assemblage and grain-supported texture. Intergranular porosity is filled with micritic and blocky cement. This association represents a shallow-water, moderate to high-energy environment within the photic zone. Based on the analysis above, it is concluded that the Lumazahi area represents a shallow marine environment within the euphotic zone, including margin shoal, margin (back reef and reef environment), and slope settings during the late Moscovian to early Asselian. The Zhuanchang area represents a deep to shallow marine environment within the euphotic zone (Figure 11).

### 5.2. Interpretation and Comparison of Cyclothems

According to Walther’s Facies Law, laterally adjacent depositional environments stack vertically as sea levels fluctuate. For example, during periods of rising sea levels, sedimentary environments typically show a seaward shift or deepening of the water body. The periodic vertical changes in sedimentary environments observed in our study area represent distinct sedimentary cycles (Type 1 and Type 2) characteristic of the platform marginal environment. These cycles exhibit shallowing-upward sequences, similar to cyclothems documented globally [6,27,79,80,81], which range from meters to tens of meters in scale. These cycles are associated with dynamic changes linked to the advance and retreat of the Gondwana ice sheet.

Cyclothems in the Lumazhai section are composed of subtidal, shallow-marine sediments on a platform margin. The sedimentary strata are continuous and without interruptions. The cyclothems vary in thickness, which is interpreted as resulting from different accommodation spaces. Type A1 (reef-bearing cycle) is characterized by a high proportion of reefs in the total cycle thickness (Figure 9a). This phenomenon is interpreted as indicating that, during transgressive or highstand systems tracts, reefs were constructed at a high deposition rate, forming elevated topography. Subsequently, as sea levels regressed, the available deposition space gradually decreased, leading to the preferential deposition of sediments in lower-lying areas adjacent to the reefs.

The Zhuanchang section comprises two types, A3 and B, which represent shallow-water subtidal and deep-water subtidal cycles, respectively. These cycles are relatively fewer in number, interpreted as representing longer-term sea-level changes in a deep setting. The thin transgressive and thick regressive sediments within the cycles are attributed to regression-dominated glacial sea-level fluctuations.

The cyclothem depositional model can exhibit significant variation across different regional tectonic activity. The well-studied Midcontinent and Russian Europe cyclothem is situated within an uplift setting, displaying a classical transgressive–regressive cyclothem [26,27,29]. In contrast, the South China Block, formed more subdued cyclothems [30]. The major cyclothems closely correspond to those observed in the Zongdi area, where they formed on a very shallow-marine platform [30]. Given the limitations of the fusulinid paleogeographic distribution and the relatively close paleogeographic proximity of the Zongdi and Lumazhai areas in South China, this study focuses on comparing the cyclothems between these two regions.

The cycles LC1–LC4 in Lumazhai exhibit a fusulinid assemblage similar to those in Zongdi, predominantly featuring abundant *Fusulinella* and *Neostaffella*, which are characteristic of the late Moscovian. The lower cycles LC5–LC6 contain scarce fusulinids, with the first appearance of *Obsoletes* and *Protriticites* at the top of cycle 6. These cycles are likely correlative with the *Beedeina* zone of the Zongdi section, corresponding to the late Kashirian.

Cycles LC7–LC9 are characterized by the presence of *Obsoletes* and *Protriticites*, with *Montiparus* first appearing in cycle 10. This indicates a correlation between LC7–LC9 and the Krevyakinian Substage (early Kasimovian), and cycle 10 with the Khamovnikian Substage (middle Kasimovian). The lower part of cycle 13 shows sparse fusulinids, while the upper part marks the first occurrence of *Rauserites*, *Triticites*, and *Schwageriniformis*. Additionally, cycles LC13–LC14 are characterized by a considerable number of *Montiparus*, indicating that cycles LC10–LC13 are likely assigned to the Khamovnikian Substage (middle Kasimovian). The top of cycle 14 records the first occurrences of *Triticites rarvulus* and *Rauserites stuckenbergi*, marking the Kasimovian–Gzhelian boundary [48]. The upper part of cycle 13 and the lower part of cycle 14 are assigned to the Dorogomilovian Substage (late Kasimovian). Cycles LC15–LC18 are dominated by *Rauserites*, *Triticites*, *Rugosofulina*, and *Quasifusulina*, with the first occurrence of *Daixina* in cycle 19. This assemblage correlates well with those in the Zongdi area. Finally, cycle 24 is characterized by the coexistence of *Eoparafusulina* and *Pseudoschwagerina* and is assigned to the Asselian [48].

**Figure 12:**
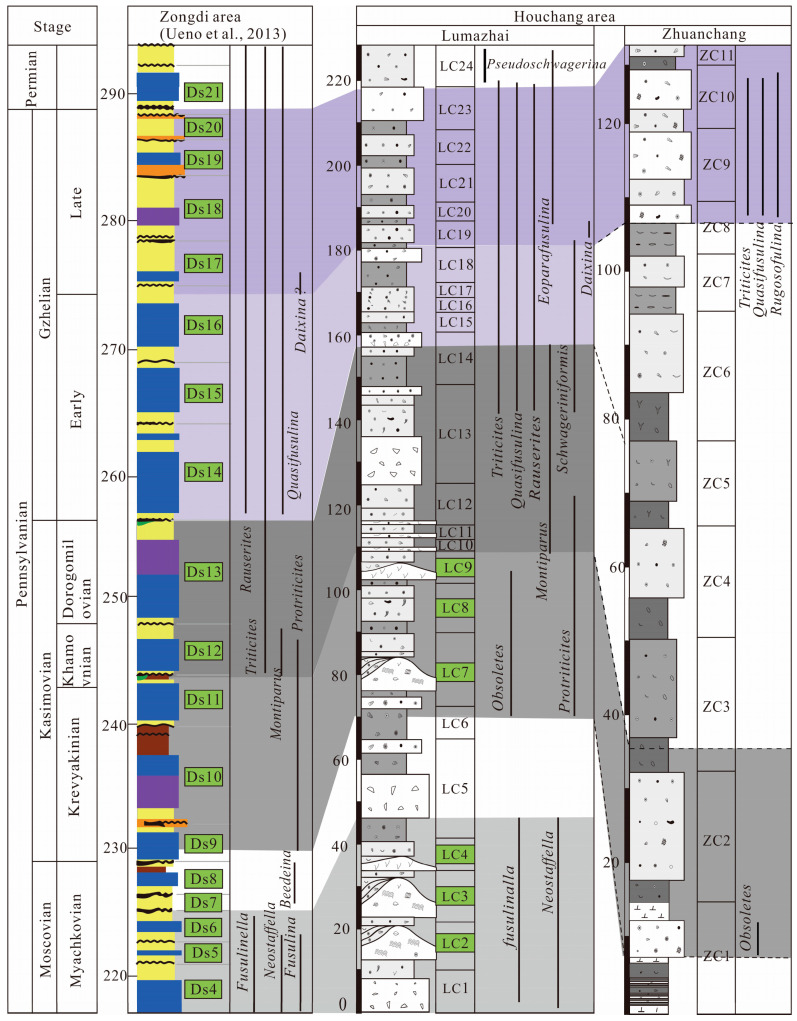
Comparison and distribution of sedimentary cycles of Zongdi, Luamzahi, and Zhuanchang sections. The shaded area showing the consistency between Type A1 (reef cycle) and Zongdi cycles [30] (modified from Ueno et al. 2012, Figure 15). Ds—Zongdi cyclothem; LC—Lumazhai cyclothem; and ZC—Zhuanchang cyclothem.

The cyclothems in Lumazhai correspond to those in the Zongdi area, and the number of cyclothems in the Lumazhai area is significantly higher than in the Zongdi area but lower in the Midcontinent. This finding suggests that Lumazhai records not only major cyclothems but also retains some minor or intermediate cyclothems (Figure 12). These cyclothems are relatively thick compared to those in the Zongdi area, presumably indicating ample accommodation space in the Lumazhai area.

### 5.3. Sea Level Change in South China

In the Late Pennsylvanian, deep-shallow settings in the South China Block recorded significant contemporaneous sea-level changes [7,12,39,82,83,84]. These changes show a clear correspondence globally with Midcontinent, strongly suggesting dynamic developments of the Gondwana ice sheet [12,82,83]. Shallow settings record high-frequency sedimentary cycles characterized by depositional unconformities, reflecting globally synchronized glacial-type sea level fluctuations [30]. The latest geochemical evidences indicate a short-term global warming event in the late Kasimovian [8,85,86]. However, corresponding sedimentological evidence supporting this warming event has not been discovered in Guizhou. Lithologic features indicate that the amplitude of sea-level fluctuations may not have exceeded hundreds of meters but rather fell within the range of tens of meters [83,87]. Here, we utilize biostratigraphy and microfacies analysis to construct a sea-level curve and discuss coupling relationship between sea-level change and paleoclimate.

**Figure 13:**
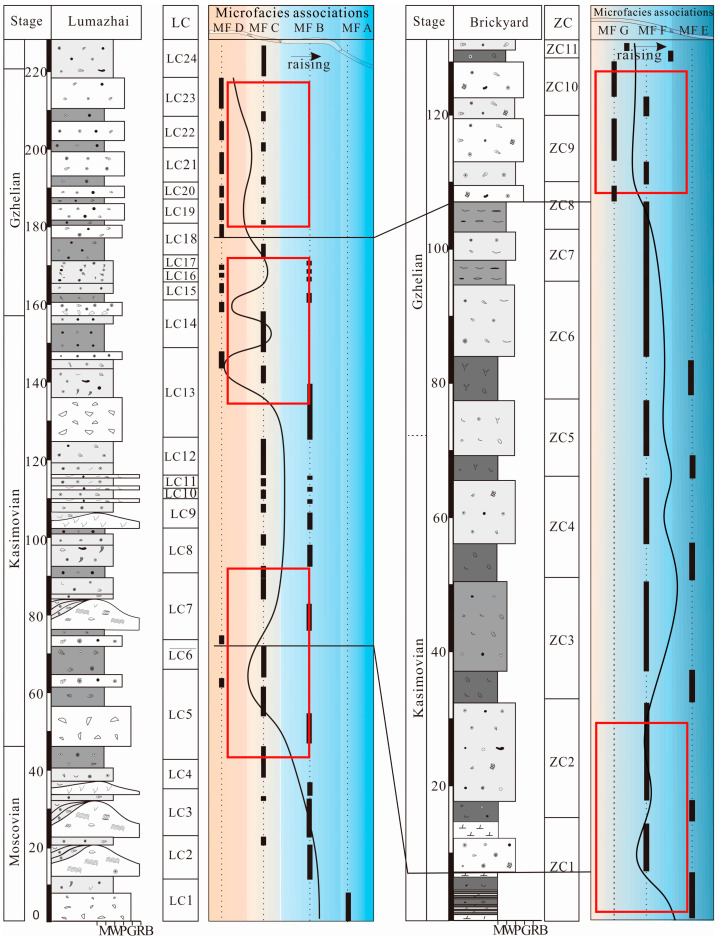
A late Moscovisn–Gzhelian stratigraphic column of the Lumazhai and Zhuanchang sections showing microfacies associations and the relative sea-level change curve. MF—microfacies association; LC–Lumazhai cyclothem; and ZC—Zhuanchang cyclothem.

During the late Moscovian to early Kasimovian, the Houchang area was characterized by brief and rapid sea level drops. In comparison to the Midcontinent and the Donets Basin, the sea-level drop in Guizhou was relatively modest, possibly due to stable tectonics and lower subsidence rates [27,29,30]. This sea-level drop may indicate glacial cooling [27]. However, the Southern Great Basin displayed an upward sea-level rise [88]. During the Kasimovian, sea level fluctuations were generally stable. Notably, there was a brief, rapid rise in the late Kasimovian, resulting in the formation of thick bioclastic wackestone with a high mudstone content in the Lumazhai section (Figure 13). Contemporaneously, coral reefs also developed in Yanbanzhai village in the Houchang area [50]. These contemporaneous records of rising sea levels are consistent with observations in the Donets basin and Midcontinent, suggesting a likely attribution to global warming [8,85,86].

In the early Gzhelian, the sea level gradually increased, characterized by the development of coral biostromes [44] and the formation of a large coral reef in corresponding areas like Bianping village [75], marking this sea-level rise. Although the rise was relatively gradual compared to Midcontinent and the Donets Basin, the consistent global trend likely indicates glacial warming [29,89]. Moving into the late Gzhelian, there was a sudden sea-level drop in the Lumazhai and Zhuanchang area. This sea-level drop corresponds to observations in the Donets Basin and Midcontinent, aligning with widespread glaciation [12,27,29,89].

The cyclothems in the Midcontinent are well known. They have been classified into three types—minor, intermediate, and major—based on the scale of transgression [26]. The cyclothems observed in Midcontinent exhibit the same magnitudes of sea-level fluctuations at the same scale [27]. Therefore, we can make a preliminary estimation of sea-level fluctuations using the reef-bearing cycles. Phylloid algal reefs serve as valuable indicators for estimating water depth for several reasons. The paleoecology of phylloid algal reefs has been determined [33,90]. Phylloid algae are considered calcareous algae closely related to *Halimeda* due to similar morphological characteristics [91,92]. Both types of bioconstructions comprise algal plates forming frameworks or mixed accumulations, exhibiting highly similar morphology, sedimentary structures, and lithofacies [92,93,94]. *Halimeda* bioherms typically develop at depths of 20–40 m, as observed in the Great Barrier Reef [93] and Indonesia [95,96]. An exception is the Miskito Channel reef, which develops at depths as deep as 40–50 m [97]. Considering the preservation condition of phylloid algal thalli, it is likely that the reefs are constructed around 40 m, while phylloid algal biostromes, due to poorly sorted thalli fragments, represent relatively shallower depths, possibly around 20 m. Low-stand conditions are characterized by shallow-water microfacies assemblages containing dasyclad green algae (typically with water depths less than 5 m) [63]. Based on the comprehensive analysis above, the sea-level fluctuations in Guizhou are estimated to be in the range of 15 to 35 m.

### 5.4. Relationship Between Reef Development Pattern and Sea-Level Fluctuations

Various bioconstructions developed in Guizhou during the Late Pennsylvanian. These bioconstructions typically exhibit thin vertical thicknesses and are overlaid by shallow-water sediments, indicating the frequent rise and fall of relative sea levels [33,40,41,42,43,44,70,74]. Although a large-scale coral reef body has developed in Bianping Village, the entire construction process was also influenced by multiple sea-level fluctuations, evident in community succession and lithofacies changes, ultimately overlain by deeper-water sediments [75]. Therefore, the development pattern of reefs in Guizhou exhibits multi-stage and cyclical construction patterns.

In the Lumazhai section, from the late Moscovian to early Kasimovian, microbial and phylloid algal reefs developed a distinctive construction pattern (Figure 12). These reef-bearing cycles show a facies-stacking sequence, with lower reef facies followed by back-reef or marginal shoal sediments. This arrangement suggests that reef facies formed during transgressive and highstand conditions, with sedimentary environments shifting toward platform margin shoals during regressions. Fusulinid biostratigraphy confirms that these cycles align with highstand cyclothems in the Zongdi section (Figure 12), indicating that intermediate- to large-scale glacio-eustatic sea-level drops caused a cessation of reef development. The lack of significant strata in the Zongdi section likely corresponds to back-reef deposits or high-frequency phylloid algal biostromes, as observed in Lumazhai. Likewise, coral reef cycles in the late Kasimovian at Yanbanzhai appear to have formed in response to major sea-level falls [50]. These large-scale, multi-phase reefs likely reflect the influence of smaller sea-level changes, later coalescing into larger reef complexes due to subsequent rapid sea-level rises [75]. Overall, reef development in Guizhou was shaped by complex glacial sea-level fluctuations operating at multiple scales.

## 6. Conclusions

Field observations and petrographic analysis identified fourteen microfacies, which were classified into seven microfacies associations. These associations represent laterally adjacent subenvironments ranging from shallow-water platform margins to adjacent deep-water basins within the Lumazhai and Zhuanchang successions. The observed vertical periodic changes in sedimentary environments are interpreted as the result of sea-level fluctuations linked to the waxing and waning of Gondwanan ice sheets.

Based on precise biostratigraphy, the sea-level changes in the study area are well correlated with those in the Midcontinent and Moscow Basin, reflecting global paleoclimate changes. The late Kasimovian saw a rapid sea-level rise, likely linked to a global warming event. Microfacies analysis suggests that sea-level fluctuations were approximately 15–35 m.

The facies changes observed in reef-bearing cycles and their comparisons indicate that sea-level fluctuations of varying magnitudes controlled a multi-phase, cyclical construction pattern of the reefs. Intermediate and major-scale sea-level fluctuations led to rapid transgressions and regressions, significantly altering sedimentary environments that influenced reef development and extinction. In contrast, small-scale changes resulted in relatively moderate alterations to sedimentary environments, which had notable effects on community succession and biostrome formation.

## Figures and Tables

**Table 1:** Microfacies of the Lumazhai section.

Microfacies	Sedimentary Structures	Description	Distribution	Depositional Environment
Intraclastic rudstone facies (F1)	Thick beds; massive deposits	Reworked, angular, and varied in size (0.5 to 7 cm in size) intraclastics and bioclasts; blocky and radial-fibrous cements (Figure 4a–c).	Distributed in bed 1	Margin slope setting
Bioclastic floatstone/rudstone facies (F2)	Thick beds; massive deposits	Reworked, angular, and poorly sorted phylloid algal fragments, corals, and brachiopod shells; blocky and radial-fibrous cements. (Figure 4d–f).	Common in Lumazhai section	Platform margin
Bioclastic packstone/grainstone facies (F3)	Thick beds; massive deposits	Poorly sorted, common foraminifers, algal fragments, bryozoan fragments, brachiopods, and corals; blocky and radial-fibrous cements. (Figure 5a,b)	Common in Lumazhai section	Platform margin
Phylloid algal boundstone facies (F4)	Tabular to lens shape	Abundant in-place phylloid algae and a few small foraminifers and brachiopods; marine cements (Figure 5c,d)	Distributed in bed 7 and bed 20	Platform margin
Microbial boundstone facies (F5)	Moundy shape	Microbial binding organisms and self-calcification; common are algae, corals, and bryozoans; marine cements (Figure 5e,f)	Distributed in some beds (3, 5 and 15)	Platform margin
Bioclastic wackestone/packstone facies (F6)	Thick beds; massive deposits	Low biodiversity, common foraminifers, fusulinids, bivalves, crinoids, and algae (Figure 6a,b)	Common in Lumazhai section	Platform margin, Back-reef environment
Intraclastic breccia facies (F7)	Thick beds; massive deposits	Reworked, angular, and poorly sorted dark-gray, bioclastic grainstone. Intergranular porosity are filled with bioclastic wackestone (Figure 6c,d)	Distributed in bed 34	Platform margin, Back-reef environment
Bioclastic grainstone facies (F8)	Thick beds; massive deposits	Subrounded and abrasive skeletal grains; blocky calcite and radiaxial-fibrous cements (Figure 6e)	Common in Lumazhai section	Margin shoal environment Above the wave base
Coated bioclastic grainstone facies (F9)	Thick beds; massive deposits	Subrounded to ellipsoid crinoid grains with micrite envelopes; calcite and radiaxial-fibrous cements (Figure 6f)	Common in some beds (31–55)	Margin shoal environment Above the wave base
Fine-grain burrowed wackestone facies (Z1)	15–45 cm thick	Common rare sponge spicules, ostracods, bryozoans, gastropods. Common burrow filled fine peloids (Figure 7a,b)	Common in Lumazhai section	Deep-water setting, Low energy environment
Spiculitic wackestone facies (Z2)	15–45 cm thick	Common angular sponge spicules in size ranging 0.3–0.8 mm, few foraminifers and ostracods (Figure 7d)	Distributed in beds 9 and bed 11	Deep-water setting Low energy environment
Mm-scale bedded bioclastic wackestone facies (Z3)	20–50 cm thick	Common bivalves, small foraminifers, ostracods. These grains exhibit a horizontal orientation (Figure 8a)	Common in Zhuancahng section	Shallow-water setting Weak energy water environment
Encrusting foraminifer wackestone facies (Z4)	20–50 cm thick	Common encrusting foraminifers, few algal, bivalve shells, crinoids, and peloids (Figure 8b)	Common in some beds (8,10 and 12)	Shallow-water setting Low energy water environment
Bioclastic packstone/grainstone facies (Z5)	40–80 cm thick	Subangular and abrasive skeletal grains, fewrare peloids and mudclasts are scarce. Locally, green algae concentrate; micritic matrix and blocky cement (Figure 8c,d)	Distributed in bottom and top of Zhuanchang section	Shallow-water setting, Below or near FWWB

## Data Availability

The original contributions presented in the study are included in the article, further inquiries can be directed to the corresponding authors.
